# Evaluation of biocontrol agents for the management of sorghum anthracnose caused by *Colletotrichum sublineola*

**DOI:** 10.3389/fpls.2025.1728722

**Published:** 2025-12-15

**Authors:** Ji-Wei Zhang, Jun-Ru Han, Fei-Fei Zhao, Xi-Rong Huang, Jin-Ling Hu, Jin-Yang Li, Kai Zhu, Lang Mao, Zi-Han Li, Jing-Yang Xia, Jie-Ting Su, Ze-Hua Liang, Xian-Gong Wang, Xian-Lin Ni, Yan-Nan Shi, Zhi-Fang Wang, Zhi-Yin Jiao, Jin-Ping Wang, Peng Lv

**Affiliations:** 1 State Key Laboratory of Crop Gene Exploration and Utilization in Southwest China, Sichuan Agricultural University, Chengdu, China; 2 Institute of Rice and Sorghum Sciences, Sichuan Academy of Agricultural Sciences, Deyang, China; 3 Institute of Millet Crops, Hebei Academy of Agricultural and Forestry Sciences, Shijizhuang, China

**Keywords:** *Sorghum bicolor*, biocontrol agents, pterostilbene, *Bacillus subtilis*, *Colletotrichum sublineola*

## Abstract

Sorghum anthracnose, caused by *Colletotrichum sublineola*, poses a severe threat to global sorghum production. In China, the rising demand for organic sorghum used in Baijiu brewing underscores the urgent need for effective biocontrol agents or microbial formulations, which remain scarce. In this study, we assessed the efficacy of six biofungicides and two microbial agents against sorghum anthracnose. All tested biocontrol agents and microbial inoculants significantly inhibited mycelial growth and spore germination of *C. sublineola in vitro*. Iron chlorin exhibited the lowest EC_50_; values for inhibition mycelial growth and spore germination, followed by pterostilbene. In greenhouse trials, Pterostilbene and Iron chlorin significantly reduced disease severity, with control efficacies of 41.3% and 51.7%, respectively, whereas *Bacillus subtilis* and *Trichoderma harzianum*achieved higher efficacies of 73.0% and 65.5%, respectively. Field trials conducted at two sites in Southwest China further confirmed pterostilbene as the most effective treatment, followed by *B. subtilis*. Collectively, our results highlight pterostilbene and *B. subtilis* as promising biocontrol agents. Their application could reduce reliance on chemical fungicides and mitigate associated environmental risks. These findings provide practical and eco-friendly strategies for anthracnose management, supporting the sustainable cultivation of organic sorghum.

## 1 Introduction

Sorghum (*Sorghum bicolor* L. Moench) is a vital global cereal crop for food, feed, and bioenergy. In China, it holds distinctive value as the primary raw material for Baijiu production, a traditional distilled liquor with profound economic and cultural significance (Zhang et al., 2025). To enhance the quality and safety of Baijiu, leading distilleries like Moutai and Luzhou Laojiao are increasingly promoting the use of organically grown sorghum in southwestern China. However, sorghum production in this region is severely threatened by anthracnose, a devastating disease caused by *Colletotrichum sublineola* that can result in yield losses exceeding 50% (Stutts and Vermerris, 2020; Yu et al., 2025). This disease not only causes severe yield losses but also compromises sorghum grain quality (Acharya et al., 2023; Fu et al., 2020; Abreha et al., 2021).

Currently, chemical fungicides represent the primary approach for managing sorghum anthracnose and other crop diseases. Agrochemicals such as carbendazim, pyraclostrobin, tebuconazole, and phenamacril are generally effective in suppressing fungal pathogens (Zhang et al., 2023). However, their overuse poses substantial risks, such as the emergence of fungicide-resistant pathogen strains, adverse impacts on soil and environmental health, the persistence of chemical residues in agricultural products, and potential threats to human safety. Growing public concern over food safety, combined with the brewing industry’s demand for organic sorghum, has accelerated the pursuit of sustainable alternatives to chemical control. Furthermore, the recurrent anthracnose epidemics in major sorghum-producing regions worldwide highlight the urgent need for the development of eco-friendly and effective management strategies.

In recent years, a diverse range of biofungicides and plant immunity inducers have been developed and applied in crop protection (Abbey et al., 2019; Tian et al., 2024). These products can be broadly classified into three categories: microbial agents, plant-derived vaccines, and natural compounds. Microbial agents such as *Bacillus subtilis* and *Trichoderma harzianum* have been widely documented to suppress fungal pathogens and enhance host immunity, with several corresponding formulations successfully commercialized (Zaim et al., 2018; Wang et al., 2019). Plant-derived elicitors include various compounds like plant-activating proteins, oligosaccharides, and salicylic acid. These substances activate plant defense mechanisms through distinct pathways: plant-activating proteins regulate metabolic processes and induce defense-related gene expression; oligosaccharides function as elicitors that trigger systemic acquired resistance; and salicylic acid, a key defense signaling molecule, modulates the expression of defense genes, maintains cell membrane integrity, and enhances antioxidant activity (Trujillo and Angulo, 2025; Mardanova et al., 2024). Abscisic acid is a potent resistance inducer known to activate over 150 defense-related genes (Qiao et al., 2023). A notable commercial application of such immunity-inducing technology is Atailing, a protein–oligosaccharide biopesticide developed by the Institute of Plant Protection at the Chinese Academy of Agricultural Sciences (Dewen et al., 2017). In addition, novel agents such as Iron chlorin, a porphyrin-based plant growth regulator, can enhance photosynthesis, root development, and stress tolerance (Zhou et al., 2024). Another example is the hypersensitive protein preparation HhrpEcc, which combines broad-spectrum disease resistance with growth promotion (Jeblick et al., 2023). Natural phenolics such as Pterostilbene, a sorghum-specific stilbene compound inducible upon anthracnose infection, further illustrate the potential of crop-derived metabolites in disease resistance (Liu et al., 2023). Similarly, erucamide, a newly identified phytoalexin, has demonstrated antifungal activity against bacterial pathogens such as *Xanthomonas oryzae* and *Ralstonia solanacearum* (Miao et al., 2025). Together, these agents highlight the diversity of natural and microbial-derived options for eco-friendly anthracnose management.

In this study, we systematically evaluated the antifungal activities of three chemical fungicides (Tebuconazole, Carbendazim, and Pyraclostrobin), six biofungicides (Iron chlorin, HhrpEcc, Alexin, S-abscisic acid, Atailing, and Pterostilbene), and two microbial agents (*B. subtilis* and *T. harzianum*) for the control of sorghum anthracnose. Using integrated *in vitro* and *in vivo* assays, we evaluated their capacities to inhibit mycelial growth, spore germination, and disease progression. Our results identify highly effective biocontrol alternatives, offering practical solutions to minimize chemical inputs, secure the production of organic brewing sorghum, and advance sustainable disease management in China’s Baijiu industry.

## 2 Materials and methods

### 2.1 Pathogen isolation and identification

Typical anthracnose-infected sorghum leaves were collected from Da’an District, Zigong City, Sichuan Province, China, in 2024. The leaf samples were surface-sterilized by sequential immersion in 75% ethanol for 30 s and 1% sodium hypochlorite for 1 min, followed by three rinses with sterile distilled water. Small pieces (approximately 5 mm²) of sterilized tissue from the lesion margins were plated on potato dextrose agar (PDA) medium and incubated at 28°C for 3 days in the dark. Emerging fungal colonies were purified by subculturing on fresh PDA. Conidial morphology was examined under a light microscope (Olympus BX53, Japan) at 400× magnification. For molecular identification, genomic DNA was extracted from 7-day-old mycelial cultures using a commercial fungal DNA extraction kit (Solarbio, Beijing, China) following the manufacturer’s instructions. The internal transcribed spacer (ITS) region of the ribosomal DNA was amplified using the universal primers ITS1 (5’-TCCGTAGGTGAACCTGCGG-3’) and ITS4 (5’-TCCTCCGCTTATTGATATGC-3’) (White et al., 1990). PCR reactions were performed in a 25 μL volume containing 12.5 μL 2× Taq Master Mix (Vazyme, Nanjing, China), 1 μL each primer (10 μmol/L), 1 μL template DNA (50 ng/μL), and 9.5 μL ddH_2_O. The thermal cycling protocol comprised an initial denaturation at 94°C for 5 min; 35 cycles of denaturation 94°C for 30 s, annealing at 55°C for 30 s, and extension at 72°C for 1 min; followed by a final extension at 72°C for 10 min. The resulting PCR products were sequenced bidirectionally by Sangon Biotech (Shanghai, China). Sequences were analyzed using BLASTn against the NCBI GenBank database for species identification. The isolated strain, designated ZG-DA-20, was preserved on PDA slants at 4°C for subsequent studies.

### 2.2 Pathogenicity confirmation

Pathogenicity of the isolated strain ZG-DA-20 was verified in accordance with Koch’s postulates. Healthy sorghum seedlings (cv. ‘GJH’, susceptible to anthracnose) at the 5-leaf stage were inoculated with a conidial suspension (1 × 10^6^ conidia/mL) prepared in sterile distilled water with 0.1% Tween-20. The suspension was sprayed onto leaves until runoff using a handheld atomizer. Control plants were treated similarly with sterile distilled water containing 0.1% Tween-20. All inoculated plants were maintained in a growth chamber at 28°C with 90% relative humidity and a 12-h photoperiod for 7 days. The pathogen was subsequently re-isolated from the developing lesions on the inoculated leaves and confirmed to be identical to the original strain ZG-DA-20 through both morphological and molecular characterization.

### 2.3 Biocontrol agents

Eleven agents were evaluated for their efficacy in controlling sorghum anthracnose caused by *C. sublineola* strain ZG-DA-20 (**Supplementary Table S1**). Three chemical fungicides served as positive controls: Pyraclostrobin (250 g/L EC, BASF Crop Protection, Jiangsu, China), Carbendazim (50% WP, Anhui Guangxin Agrochemical Co., Ltd., China), and Tebuconazole (430 g/L SC, Jiangsu Qizhou Green Chemical Co., Ltd., China). Six biofungicides were tested: S-abscisic acid (5% SL, Sichuan Longmang Fusheng Technology Co., Ltd., China), Alexin (0.3% Matrine AS, Xiangyu Agricultural Technology Co., Ltd., China), Atailing (0.5% Physcion AS, Hebei Zhongbao Lunong Crop Technology Co., Ltd., China), Iron chlorin (also known as dihydroporphyrin iron, 0.5% SL, Anqing Baite Biological Engineering Co., Ltd., China), HhrpEcc (0.5% Osthole AS, Sichuan Haiboshi Biological Technology Co., Ltd., China), and Pterostilbene (99.5% SC, Beijing Solebo Technology Co., Ltd., China). Additionally, two microbial antagonists were incorporated: *Bacillus subtilis* (10^10^ CFU/g WP, Hebei Guanlong Agrochemical Co., Ltd., China) and *Trichoderma ha0rzianum* (2 × 10^9^; CFU/g WP, Hubei Qiming Biological Engineering Co., Ltd., China). All agents were initially prepared as stock solutions according to the field concentrations recommended by the manufacturers, followed by serial dilutions. Based on these tests and preliminary *in vitro* experiments, we determined the final concentration ranges (**Supplementary Table S2**) used for EC_50_; estimation (Abo-Zaid et al., 2025). Sterile distilled water served as the negative control throughout the study.

### 2.4 Mycelial growth inhibition assay

Stock solutions of each agent were serially diluted to obtain a graded concentration series, as determined by preliminary range-finding tests. One milliliter of each diluted solution was mixed with 99 mL of molten potato sucrose agar (PSA) medium (cooled to 45-50°C) to prepare agent-amended plates. Control plates received 1 mL of sterile distilled water. Mycelial plugs (5 mm diameter) were excised from the actively growing margins of 7-day-old ZG-DA-20 colonies on PDA and placed centrally on the amended PSA plates. Plates were incubated at 28°C for 6 to 8 days in the dark. Colony diameters were measured using the cross-measurement method (two perpendicular diameters, subtracting the plug diameter). Each treatment consisted of four times. Mycelial growth inhibition rate was according to the following formula (Lü et al., 2023):


Inhibition rate(%)=[(Dc−Dt)/Dc]×100

where Dc is the average colony diameter on control plates, and Dt is the average colony diameter on treatment plates.

### 2.5 Conidial germination assay

Stock solutions of each agent were diluted to the desired concentrations and incorporated into water agar (WA) medium at a 1% (v/v) ratio. Control plates were prepared by adding an equivalent volume of sterile distilled water. Conidial suspensions of ZG-DA-20 were prepared by flooding 7-day-old PDA cultures with sterile distilled water containing 0.1% Tween-20, scraping the surface, and filtering through double-layer cheesecloth. The concentration was adjusted to 1 × 10^6^ conidia/mL using a hemocytometer. Fifty microliters of the suspension were spread evenly onto each WA plate. Plates were incubated at 28°C for 9 h in darkness. Germination was assessed microscopically by examining 100 randomly selected conidia per plate; a conidium was considered germinated if the germ tube length exceeded half the conidial length. Each treatment was replicated four times. Conidial germination rate was calculated as follows:


Germination rate (%)=(Number of germinated conidia/Total conidia observed)×100

Inhibition rate (%) was then determined as (Hu et al., 2021):


Inhibition rate (%)=[(Gc-Gt)/Gc]×100

where Gc is the germination rate in the control, and Gt is the germination rate in the treatment.

Virulence regression equations were constructed using the agent concentration as the x-axis and the probit-transformed inhibition rate as the y-axis. Regression parameters, correlation coefficients (r), and median effective concentrations (EC_50_;) with 95% confidence intervals were estimated using probit analysis in SPSS 26.0 software. EC_50_; values were used to compare inhibitory potencies and select agents with high efficacy at low doses.

### 2.6 Greenhouse efficacy trials

Sorghum seeds (cv. ‘GJH1’) were sown in plastic pots (15 cm diameter) filled with sterilized potting mix (peat:vermiculite:perlite = 2:1:1, v/v/v) and grown in a greenhouse at 28 °C with a 12-h photoperiod. At the six-leaf stage, uniform seedlings were selected and transferred to an inoculation chamber maintained at approximately 75% relative humidity. To sustain high humidity, the chamber was covered with white plastic film. Plants were inoculated by spraying their leaves with a conidial suspension of ZG-DA-20 (1 × 10^6^ conidia/mL in 0.1% Tween-20) until runoff occurred. After 36 h, agents were applied at their respective effective concentrations (from *in vitro* assays) using a handheld sprayer. The experimental design comprised the following treatments: sterile water (blank control), chemical fungicides (positive controls), and pathogen inoculation without agent (negative control). Each treatment had 10 pots with 3 plants per pot, arranged in a completely randomized design. After 7 days, plant growth parameters (fresh weight, root length, and plant height) were measured using a digital balance and ruler. Specifically, root length was measured as the distance from the stem base to the tip of the longest root using a ruler, with each plant measured individually. Mean values for each treatment were used for analysis. Disease severity was rated on a 1–5 scale (Zhang et al., 2025): 1 = no symptoms (healthy); 2 = hypersensitive lesions on lower leaves; 3 = acervuli on bottom leaves; 4 = acervuli on middle to bottom leaves; 5 = acervuli on whole plant including flag leaf. Disease index (DI) was calculated as:


DI=[Σ(Rating×Number of plants at that rating)/(Maximum rating×Total plants)]×100

Control efficacy (%) was determined as (Egel et al., 2019):


Control efficacy (%)=[(DI negative control-DI treatment)/DI negative control]×100

### 2.7 Field efficacy trials

Field trials were conducted during May 2025 in experimental sorghum fields in Chouzhou (103.66°E, 30.57°N) and Luhzhou (105.35°E, 28.96°N), Sichuan Province, China. Sorghum plants (cv. ‘GJH1’) were cultivated at a spacing of 40 cm × 20 cm in plots of 20m², arranged in a randomized complete block design with four replicates per treatment. Five selected agents (Atailing, Iron chlorin, *T. harzianum*, pterostilbene, and *B. subtilis*) were tested, along with a water control, resulting in 50 plots (2 plants per plot). A conidial suspension of ZG-DA-20 (1 × 10^6^ CFU/mL) was applied to leaves using a backpack sprayer, followed 30 min later by agent application at manufacturer-recommended field concentrations. Standard agronomic practices were followed, without additional fungicide applications. For field evaluations, however, the rating standards were manually adjusted by taking into account local meteorological data (rainfall and average daily temperature) recorded after inoculation, the rate of disease progression, and the proportional distribution of severity levels observed under controlled greenhouse conditions. Fourteen days post-inoculation, disease incidence (percentage of infected plants), severity (1–5 scale as in greenhouse), and disease index were recorded for all plants per plot. Control efficacy was calculated as in the greenhouse trials. Grading criteria for anthracnose severity were adjusted based on natural field conditions, incorporating environmental factors such as rainfall and temperature.

### 2.8 Statistical analysis

All data were analyzed with SPSS 26.0 software (IBM Corp., Armonk, NY, USA). Means of plant growth parameters, inhibition rates, EC_50_; values, disease indices, and control efficacies were compared using one-way analysis of variance (ANOVA) followed by the least significant difference (LSD) test at P< 0.05.

## 3 Results

### 3.1 Pathogen isolation and identification

**Figure 1:**
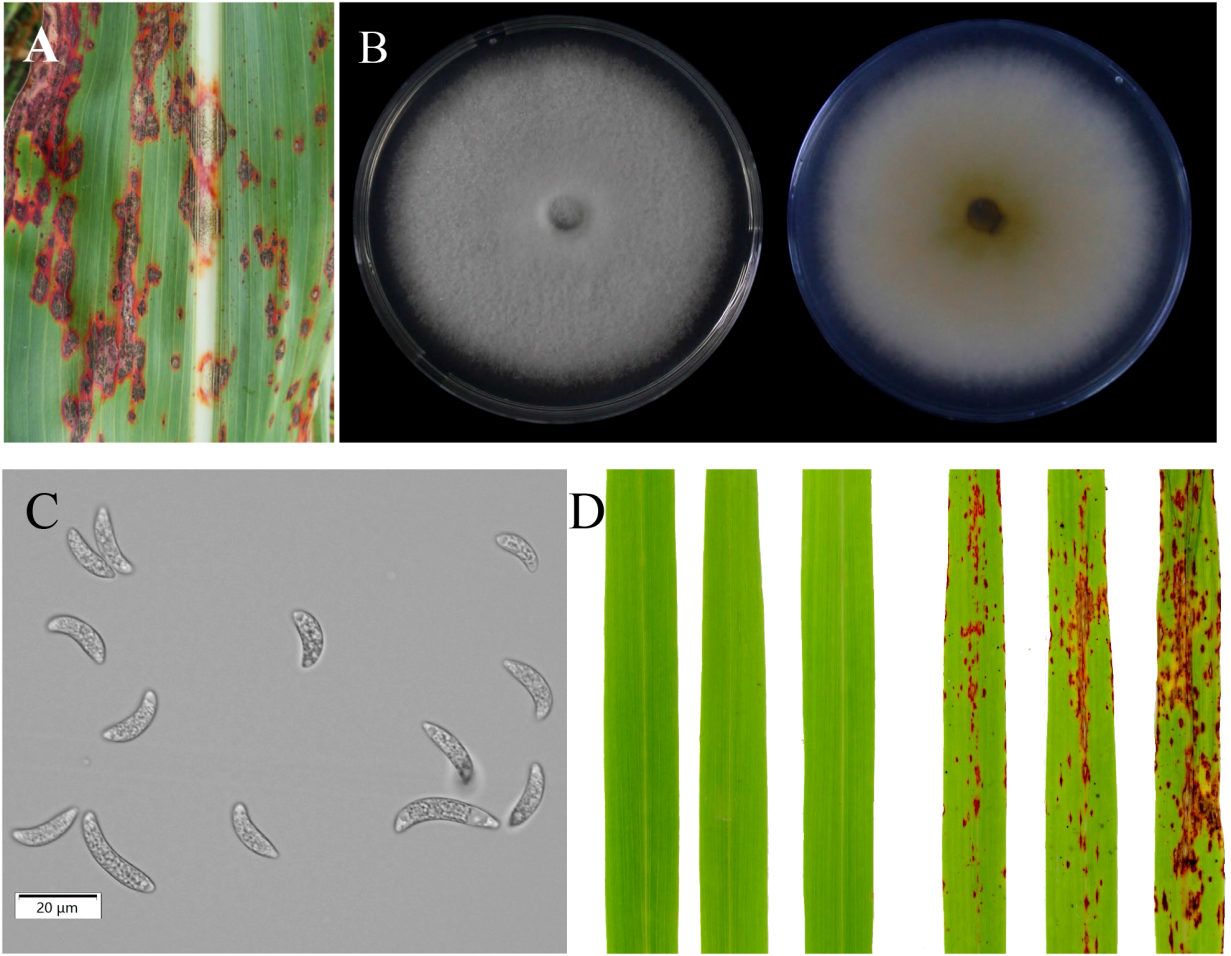
Isolation and identification of *C*. *sublineola*. **(A)** Field symptoms of sorghum anthracnose caused by *C. sublineola* isolate ZG-DA-20. **(B)** Colony morphology of ZG-DA-20. **(C)** Conidial morphology of ZG-DA-20. **(D)** Pathogenicity assessment of ZG-DA-20 on sorghum, comparing a mock-inoculated control plant (left, sprayed with water) with a pathogen-inoculated plant (right) at 7 days post inoculation.

A highly virulent fungal strain of *C. sublineola*, designated ZG-DA-20, was isolated from symptomatic sorghum leaves collected in Da’an District, Zigong City, in southwestern China. The disease symptoms and morphological characteristics of the pathogen confirmed its identity as *C. sublineola* (**Figure 1A**). When cultivated on PDA at 28°C for 7 days, the colonies initially exhibited a white coloration that gradually turned gray, forming abundant acervuli with setae (**Figure 1B**). The conidia were crescent-shaped, hyaline, and aseptate, measuring 15-25 μm in length and 3-5 μm in width, aligning with previously described characteristics for *C. sublineola* (**Figure 1C**). Molecular identification based on the ITS region showed that the sequence of ZG-DA-20 (539/539 bp) was 100% identical to the reference strain *C. sublineola* GD202206 (GenBank: ON680862.1). Pathogenicity was then confirmed via Koch’s postulates. Sorghum seedlings inoculated with a conidial suspension developed characteristic anthracnose symptoms, including red-brown lesions bearing acervuli at 7 dpi. In contrast, mock-inoculated control plants remained healthy and symptom-free (**Figure 1D**). The same fungus was successfully re-isolated from the diseased tissues, thereby fulfilling Koch’s postulates and confirming *C. sublineola* as the causal agent of the observed symptoms.

### 3.2 *In vitro* inhibition of mycelial growth rate and germination of conidium

**Figure 2:**
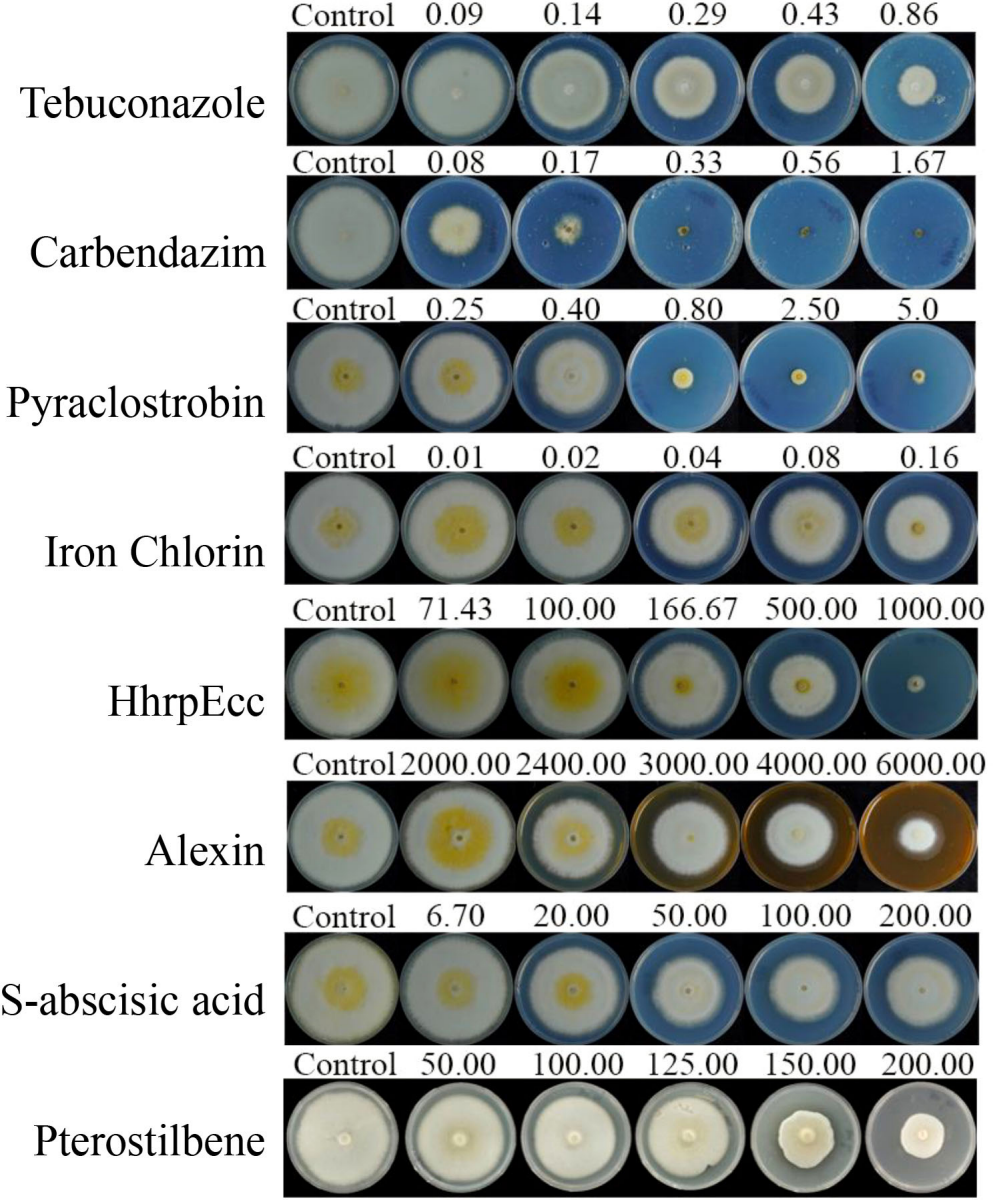
Inhibitory effects of three chemical fungicides and five biofungicides on the mycelial growth of *C. sublineola*. Fungicides tested: Tebuconazole, Carbendazim, Pyraclostrobin, Iron chlorin, HhrpEcc, Alexin, S-abscisic acid, and Pterostilbene.

The three chemical fungicides and five biofungicides (Iron chlorin, HhrpEcc, Alexin, S-abscisic acid, and Pterostilbene) exhibited varying degrees of inhibitory activity against the mycelial growth and spore germination of *C. sublineola in vitro* (**Figures 1**, **2**; **Tables 1**, **2**). For mycelial growth inhibition, all agents significantly reduced growth compared with the control, with the three chemical agents showing particularly strong inhibition (**Figures 1**, **2**). Among the five biofungicides, iron chlorin was the most effective, with the lowest median effective concentration (EC_50_ = 0.0790 µg/mL), followed by Pterostilbene (EC_50_ = 113.3241 µg/mL). Notably, HhrpEcc displayed a steep regression slope (k = 3.2209), indicating a rapid decline in pathogen viability with increasing concentrations. The correlation coefficients (r) ranged from 0.9113 to 0.9969, implying that all agents exhibited significant dose-response relationships (**Table 1**).

**Table 1:** Toxicity of three chemical fungicides and five biofungicides on the mycelial growth of *C. sublineola*.

Pesticide name	Toxicity regression equation	EC_50_(μg/mL)	Correlation coefficient	P-value
Iron chlorin	Y=1.94X+7.14	0.08	0.9831	0.0026
Carbendazim	Y=3.64X+8.45	0.11	0.9406	0.0172
Pyraclostrobin	Y=1.16X+5.77	0.22	0.9446	0.0155
Tebuconazole	Y=1.85X+5.34	0.66	0.9942	0.0005
Pterostilbene	Y=2.54X+6.87	113.32	0.9113	0.0020
S-abscisic acid	Y=1.16X+2.58	121.88	0.9268	0.0235
HhrpEcc	Y=3.22X-2.56	223.10	0.9969	0.0002
Alexin	Y=2.85X-5.53	5017.60	0.9520	0.0125

**Table 2:** Toxicity of three chemical fungicides and five biofungicides on the spore germination of *C. sublineola*.

Pesticide name	Toxicity regression equation	EC_50_(μg/mL)	Correlation coefficient	P-value
Iron Chlorin	Y=1.11X+6.46	0.05	0.9619	0.0089
Carbendazim	Y=1.65X+6.22	0.18	0.9689	0.0065
Pyraclostrobin	Y=4.09X+6.34	0.47	0.9585	0.0101
Pterostilbene	Y=5.78X+10.64	65.39	0.9939	0.0030
Tebuconazole	Y=2.39X+1.25	37.06	0.9171	0.0283
S-abscisic acid	Y=0.83X+3.47	68.01	0.9956	0.0003
HhrpEcc	Y=4.46X-4.11	110.70	0.9586	0.0100
Alexin	Y=2.87X-5.87	6061.69	0.9967	0.0002

Spore germination assays yielded results consistent with those observed for mycelial growth inhibition. Among the five biofungicides tested, iron chlorin again exhibited the strongest inhibitory effect, showing with the lowest EC_50_; (0.0475 µg/mL), followed by pterostilbene (65.3901 µg/mL). HhrpEcc and S-abscisic acid also displayed moderate effects, with EC_50_; values of 110.7003 and 68.0103 µg/mL, respectively (**Table 2**). Notably, the EC_50_; values for spore germination were consistently lower than those for mycelial growth across all tested agents.

### 3.3 Greenhouse experiments

**Figure 3:**
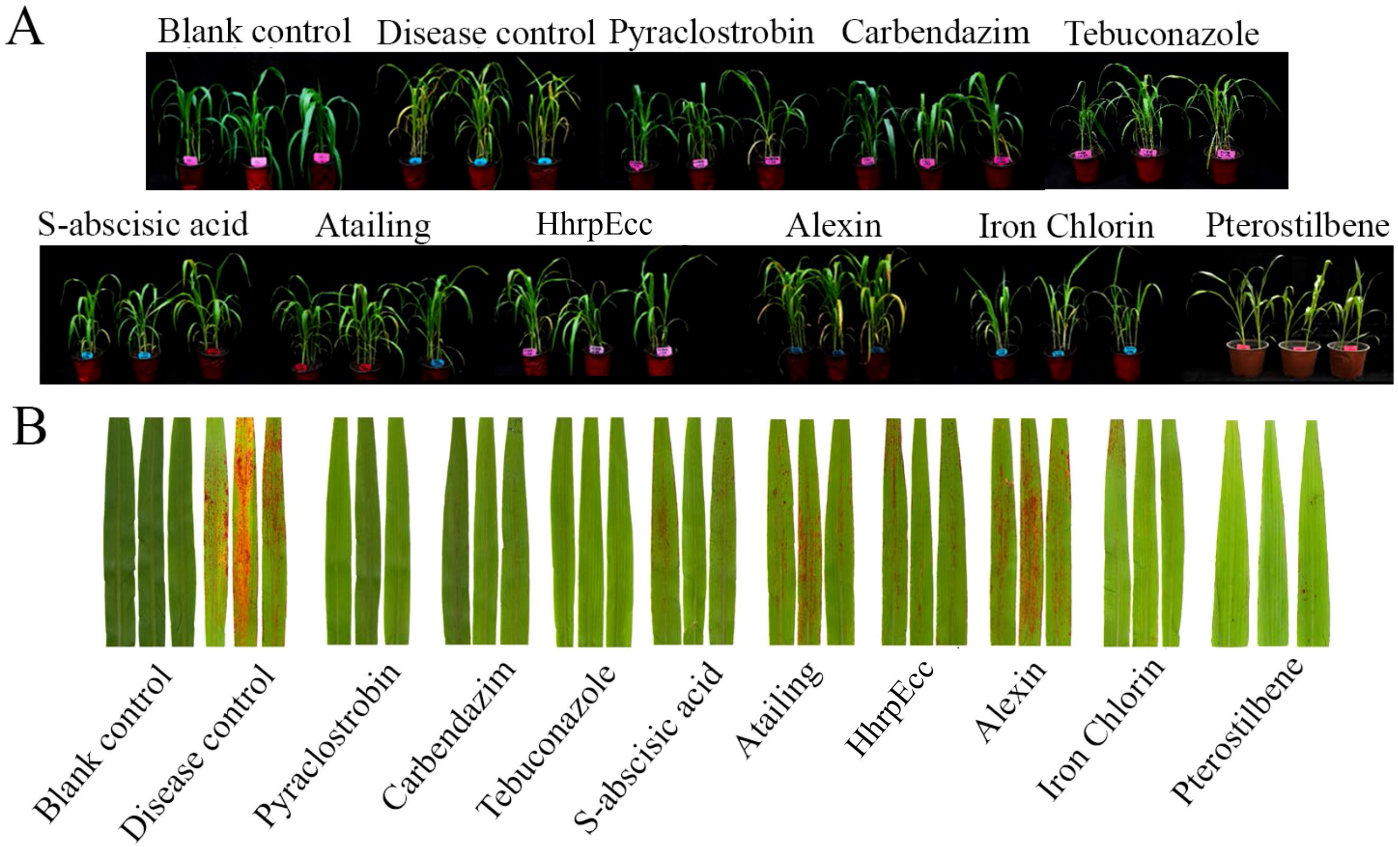
Control efficacy of three chemical fungicides and five biofungicides against sorghum anthracnose in greenhouse experiments. **(A)** Pot experiment showing effects of different treatments on sorghum plant growth and anthracnose. Blank control: sprayed with sterile water, no *C*. *sublineola* inoculation; Disease control: *C*. *sublineola* inoculated, no fungicide treatment. **(B)** Leaf anthracnose symptoms following treatment with different fungicides.

In greenhouse trials, all agents reduced anthracnose severity compared with the inoculated control, which exhibited a disease index of 88.89 and extensive leaf lesions covering over 70% of the leaf area (**Figure 3**; **Table 3**). Among the six test biocontrol agents, pterostilbene and iron chlorin provided the most effective protection, achieving disease indices of 51.85 and 42.96, respectively, and preventing the formation of visible red spots on leaves (**Figure 3**). These results corresponded to control efficacies of 41.3% for pterostilbene and 51.7% for Iron chlorin. Moderate efficacy was observed for HhrpEcc (33.8% efficacy; disease index 58.89), S-abscisic acid (36.7%; 56.30), and Atailing (25.8%; 65.93). Alexin showed the lowest control efficacy (16.7%; 87.40) (**Table 3**). Regarding plant growth parameters, none of the treatments had a significant impact on plant height or root length. As expected, pathogen inoculation significantly reduced fresh weight, indicating that anthracnose significantly inhibits plant growth. After treatment with biocontrol agents, only pterostilbene restored plant fresh weight to a level statistically comparable to that of the healthy mock-inoculated control, while other treatments still significantly reduced plant fresh weight (**Table 3**).

**Table 3:** Control efficacy of three chemical fungicides and six biofungicides against sorghum anthracnose.

Pesticide name	Plant height/cm	Root length/cm	Fresh weight/g	Disease index	Efficacy/%
Blank control	55.52 + 3.93a	29.57 + 5.65a	4.44 + 1.42a	/	/
Disease control	56.37 + 4.39a	32.29 + 2.66a	2.42 + 0.36b	88.89 + 11.11a	/
Carbendazim	58.13 + 5.49a	20.68 + 3.3b	3.95 + 1.51a	31.11 + 3.85c	65.00
Pyraclostrobin	56.14 + 3.13a	19.29 + 0.45c	3.61 + 1.13ab	23.33 + 6.19b	73.80
Tebuconazole	48.77 + 4.40b	29.19 + 2.14a	1.92 + 0.35b	30.37 + 2.57b	65.80
S-abscisic acid	54.74 + 1.06a	28.12 + 1.00a	2.22 + 0.02b	56.30 + 15.41b	36.70
Alexin	56.24 + 2.16a	30.3 + 1.79a	2.44 + 0.67b	87.40 + 1.28ab	16.70
HhrpEcc	53.95 + 2.64a	28.00 + 2.21a	2.17 + 0.27b	58.89 + 2.94b	33.80
Iron Chlorin	51.16 + 3.71ab	32.67 + 4.80a	2.17 + 0.48b	42.96 + 20.65b	51.70
Atailing	55.21 + 1.24a	26.03 + 0.74a	2.13 + 0.19b	65.93 + 11.18b	25.80
Pterostilbene	58.30+0.59a	29.41+3.97a	4.19+0.17a	51.85+1.01b	41.30

**Figure 4:**
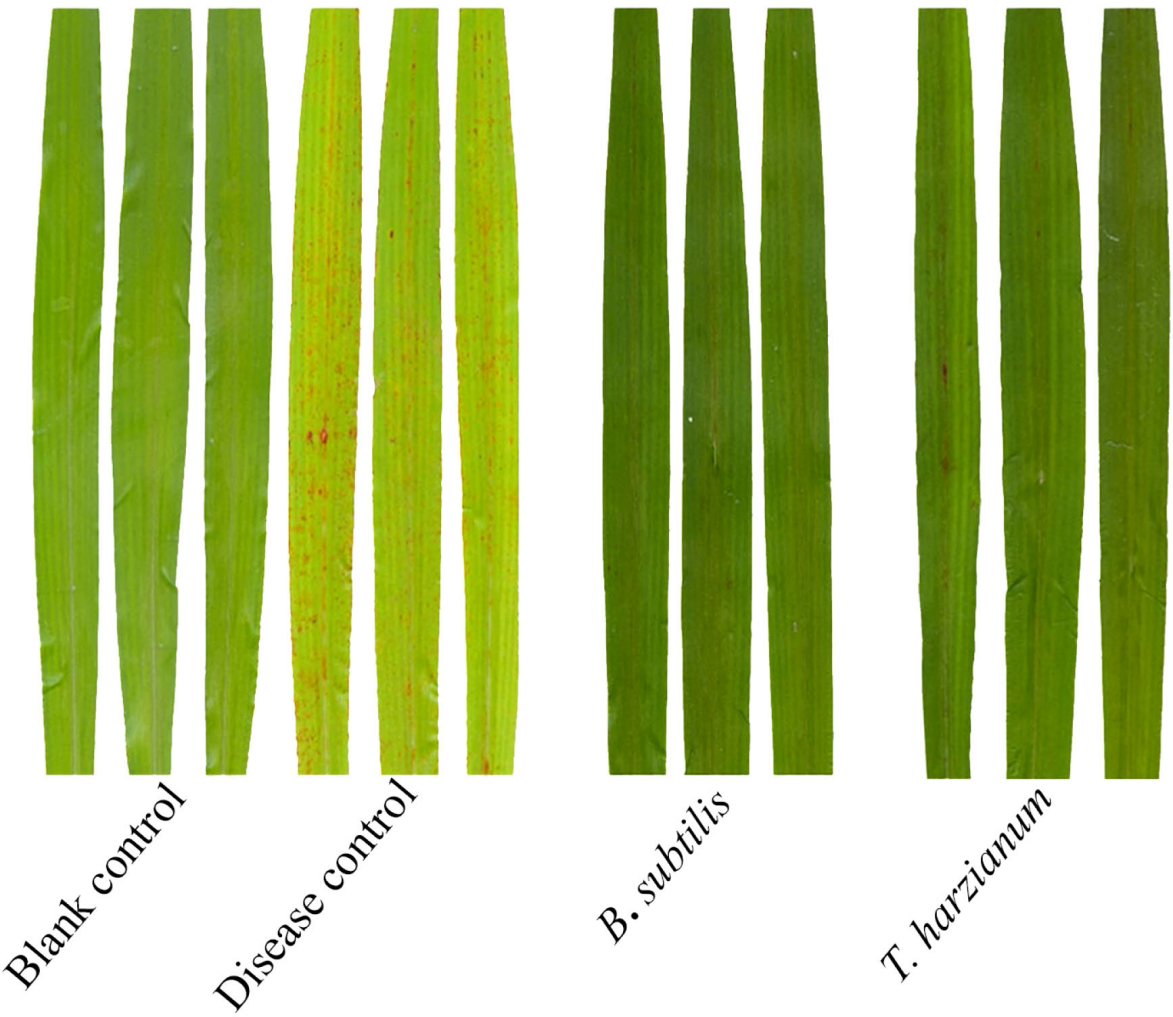
Control effects of *B*. *subtilis* and *T. harzianum* against sorghum anthracnose in greenhouse.

A separate greenhouse pot experiment was also conducted to evaluate the biocontrol efficacy of *T. harzianum* and *B. subtilis* against sorghum anthracnose. At 7 dpi after foliar application of spore suspensions at the manufacturer-recommended concentrations, *B. subtilis* and *T. harzianum* exhibited high relative control efficacies of 73.0% and 65.5%, respectively (**Table 4**). To further investigate their modes of action, dual-culture assays were performed, showing that *T. harzianum* and *B. subtilis* significantly inhibited the mycelial growth of strain ZG-DA-20, leading to restricted colony expansion (**Figure 4**). These results demonstrate that *T. harzianum* and *B. subtilis* achieve effective control through direct antagonism and possibly by inducing host resistance. Their demonstrated performance underscores the potential of these microbial agents as sustainable alternatives to chemical fungicides, which could help reduce pesticide residues and mitigate the risk of resistance development.

**Table 4:** Control efficacy of *B. subtilis* and *T. harzianum* against sorghum anthracnose.

Pesticide name	Plant height/cm	Root length/cm	Fresh weight/g	Disease index	Efficacy/%
Blank control	45.20 + 1.85a	17.56 + 1.44b	2.51 + 0.13a	–	–
Disease control	46.61 + 2.21a	21.72 + 2.82a	1.85 + 0.30b	82.35 + 0.93a	–
Bacillus subtilis	53.70 + 0.54a	15.36 + 0.99b	2.38 + 0.20ab	22.22 + 3.70b	73.00
Trichoderma harzianum	47.17 + 4.14a	13.34 + 1.24c	2.25 + 0.27ab	28.40 + 5.66b	65.50

### 3.4 Field experiments

**Figure 5:**
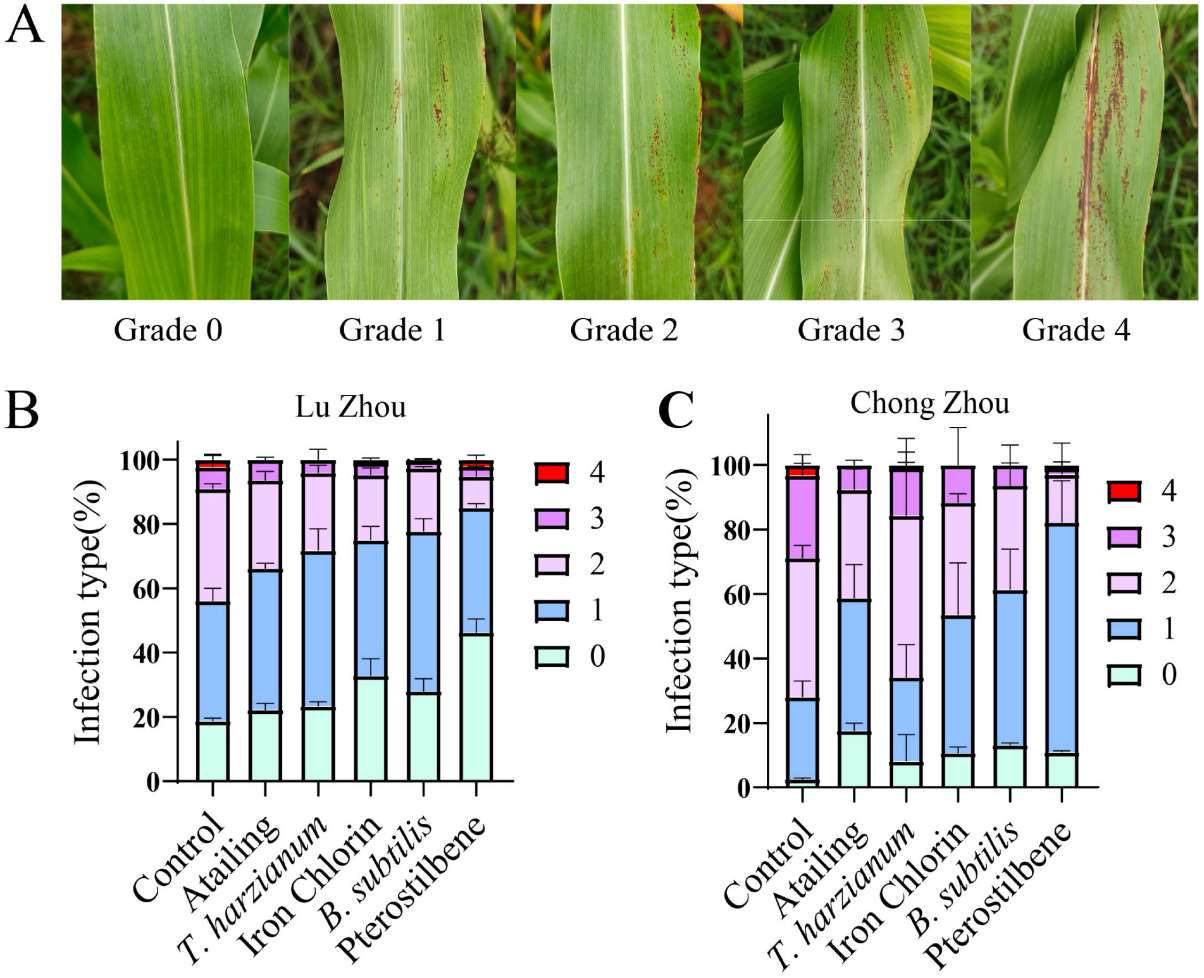
Disease severity grades and incidence of sorghum anthracnose in field trials. **(A)** Disease severity scoring chart used for filed assessment. Disease severity was scored on a scale of 0 to 4 (0 = no visible infection; 1 = very light infection; 2 = light infection; 3 = moderate infection; 4 = severe infection). **(B, C)** Distribution of disease-severity levels in Luzhou **(B)** and Chongzhou **(C)**.

Field trials conducted in Luzhou and Chongzhou confirmed the consistency of greenhouse results, with pterostilbene and *B. subtilis* providing consistent control across locations. Disease severity on sorghum leaves was assessed using a 0–4 grading scale, where 0 represented no symptoms and 4 indicated severe lesions covering most of the leaf surface (**Figure 5**). This grading system highlights progressive deterioration with increasing infection severity. Across the two trial sites, pterostilbene-treated plants exhibited fewer instances of grades 2-4, indicating generally milder disease severity, followed by those treated with *B. subtilis*, which showed a similar but slightly less pronounced reduction in higher-grade symptoms (**Figure 5**).

In Luzhou, the inoculated control treatment showed a disease incidence of 81.24% and a disease index of 34.1 (**Table 5**). Application of pterostilbene reduced the disease incidence to 53.69% and the disease index to 18.98, achieving 44.33% efficacy. *B. subtilis* achieved 27.43% efficacy, with incidence disease at 71.93% and disease index at 23.89. Iron chlorin and Atailing achieved 26.78% and 19.9% efficacy, respectively. In Chongzhou, where disease pressure was higher, with the inoculated control showing a disease incidence of 97.44% and a disease index of 50.1. However, pterostilbene treatment excelled again at 45.01% efficacy and a disease index of 27.5, followed by *B. subtilis* at 34.20% efficacy and disease index of 32.7, then Atailing at 34.48% efficacy and disease index of 32.8. *T. harzianum* demonstrated limited efficacy at 13.28% (**Table 5**). Statistical analysis confirmed significant differences among treatments, with pterostilbene and *B. subtilis* outperforming the others in both locations, as evidenced by the skewed distributions toward lower severity grades in the stacked bar charts.

**Table 5:** Disease incidence, disease index, and control efficacy of five selected biological or microbial agents against sorghum anthracnose.

Pesticide name	Disease incidence/%		Disease index		Efficacy/%	
	Luzhou	Chongzhou	Luzhou	Chongzhou	Luzhou	Chongzhou
Control	81.24 ± 1.59a	97.44 ± 0.54a	34.10 ± 4.83a	50.1 ± 0.85a	/	/
Atailing	77.73 ± 3.30ab	82.42 ± 3.43ab	29.49 ± 2.64ab	32.8 ± 2.40bc	12.91	34.48
Trichoderma harzianum	76.64 ± 2.32ab	91.80 ± 11.59b	27.21 ± 3.08ab	43.5 ± 7.07ab	19.96	13.28
Iron Chlorin	67.15 ± 9.08b	89.29 ± 2.53ab	24.43 ± 3.33bc	37.5 ± 7.64abc	26.78	25.01
Bacillus subtilis	71.93 ± 6.63ab	86.89 ± 0.87ab	23.89 ± 2.43bc	32.7 ± 7.35bc	27.43	34.20
Pterostilbene	53.69 ± 7.22c	89.08 ± 0.56ab	18.98 ± 5.38c	27.5 ± 5.37c	44.33	45.01

## 4 Discussion

This study comprehensively evaluated the potential of various biocontrol agents and microbial formulations as sustainable alternatives to chemical fungicides for managing sorghum anthracnose, a disease caused by *C. sublineola* that threatens organic sorghum production for China’s Baijiu industry. Integrating *in vitro* assays, greenhouse trials, and field experiments, we systematically assessed the antifungal activities and practical applications of these agents against the virulent strain ZG-DA-20. We hypothesized that certain bio-based strategies could provide disease control comparable to synthetic fungicides, potentially through combined mechanisms involving direct antagonism and the induction of host resistance.

Our study indicates that all tested agents inhibited the mycelial growth and spore germination of *C. sublineola in vitro*. Iron chlorin showed the strongest inhibitory effect, with the lowest EC_50_; values recorded (0.0790 µg/mL for mycelial growth and 0.0475 µg/mL for spore germination), followed closely by pterostilbene (**Figure 2**; **Tables 1**, **2**). In greenhouse trials, pterostilbene and iron chlorin delivered control efficacies of 41.3% and 51.7%, respectively, while the microbial antagonists *B. subtilis* and *T. harzianum* outperformed them with efficacies of 73.0% and 65.5% (**Figures 3**, **4**; **Tables 3**, **4**). Field trials across two sites in Southwest China (Luzhou and Chongzhou) highlighted pterostilbene as the most consistent performer, averaging 44.67% efficacy, with *B. subtilis* at 30.82% (**Figure 5**; **Table 5**). The greenhouse experiments emphasize mechanistic insights and potential resistance, while field trials reflect practical performance and stability. These findings align with previous reports indicating that beneficial microorganisms often perform better under controlled greenhouse conditions than in field environments, likely due to reduced survival and colonization under variable natural conditions (Hu et al., 2021; Luo et al., 2025). While, pterostilbene is more stable under natural environmental conditions. In addition, the induced defense responses of the host may synergize with pterostilbene, thereby enhancing the overall disease control efficacy (Chan et al., 2019). Pterostilbene exhibited potent antifungal activity against multiple pathogens, suggesting its strong potential as an eco-friendly alternative for postharvest disease control (Xu et al., 2018). Notably, plants treated with pterostilbene also exhibited enhanced growth metrics compared to those treated with other biocontrol agents (**Table 4**), underscoring that this compound not only suppresses disease but also supports plant development without adverse effects.

Following previous studies, we characterized the highly virulent strain ZG-DA-20 using morphological and molecular characterization of the ITS region, further reinforcing its genetic diversity and pathogenicity (Xavier et al., 2018; Chala et al., 2010). In our evaluation, selected biocontrol agents including pterostilbene and iron chlorin demonstrated substantial efficacy in both *in vitro* and greenhouse trials. The superior *in vitro* performance of iron chlorin is likely attributable to its porphyrin structure, which can induce oxidative stress in fungal cells, thereby compromising membrane integrity and metabolic pathways (Missall et al., 2004; El-Sayed et al., 2022). Similarly, pterostilbene showed strong inhibitory effects, as reflected by a steep regression slope of 5.7753 (**Table 1**), indicates potent inhibition via stilbene-mediated disruption of spore germination and mycelial expansion (Xu et al., 2022). Recent metabolomic studies have shown that anthracnose infection in sorghum significantly induces the expression of *SbSOMT*, a key gene in pterostilbene biosynthesis, leading to its accumulation in the leaves (Lui et al., 2023). Just as licorice extracts modulate defense pathways (Hermann et al., 2022), pterostilbene emerges as a highly effective natural antimicrobial compound. Collectively, these findings advance the current understanding of biocontrol strategies against *C. sublineola*, particularly in the context of organic sorghum production, where research has historically emphasized genetic resistance over microbial or compound-based interventions (Abreha et al., 2021).

The strong field performance of *B. subtili* observed in this study aligns with its documented efficacy in other pathosystems. For instance, in pepper anthracnose caused by *C. gloeosporioides*, lipopeptides produced by *B. subtilis* suppressed fungal growth by 70-80% (Ashwini and Srividya, 2014). Similarly, the greenhouse efficacy of *T. harzianum* corresponds with Indian native Trichoderma isolates that effectively control sorghum anthracnose through mycoparasitism, achieving up to 65% inhibition (Yadav et al., 2020). Although *T. harzianum* displayed strong antagonism in dual-culture assays, its inconsistent field performance suggests sensitivity to environmental factors, such as humidity and soil conditions, that may influence hyperparasitism and mycoparasitic enzyme activity (Pandey et al., 2024). Both greenhouse and field trials have demonstrated that *B. subtilis* exhibits strong biocontrol effects against the sorghum anthracnose (**Figures 4**, **5**). As supported by previous research, its biocontrol capacity involves multiple mechanisms, including lipopeptide-mediated antibiotic effects and induced systemic resistance (ISR) triggered via salicylic acid (SA) and jasmonic acid (JA) signaling pathways (Yu et al., 2022; Hansel et al., 2024). Overall, our findings support synergistic, system-level effects among microbial agents, where field efficacy depends less on *in vitro* potency and more on complex host-microbe-environment interactions that enhance biocontrol performance under natural conditions. These findings have broader relevance for managing *Colletotrichum* diseases across cropping systems, supporting the development of climate-resilient strategies that integrate biofungicides with host resistance, as recently demonstrated in Ethiopian field trials (Tadesse et al., 2024).

Several limitations should be considered when interpreting the findings of this study. First, the evaluation focused on a single virulent strain (ZG-DA-20), which may not fully represent the pathotype diversity reported in *C. sublineola* populations from the United States and Africa (Prom et al., 2014; Zeleke et al., 2024). Second, methodological constraints hindered the exploration of long-term field persistence, with trials limited to one season, and the absence of data on microbial colonization dynamics prevents a conclusive understanding of ISR mechanisms. Third, disease assessment relied primarily on visual severity grading, which is susceptible to observer bias, while environmental variability between the two field sites (e.g., in rainfall and temperature) may have contributed to the differences in control efficacy observed in Luzhou and Chongzhou (**Figure 5**). Finally, this study is constrained from addressing interactions with co-occurring sorghum diseases or diverse cultivars, thus limiting broader generalizability.

To address the gap identified in this study, researchers may consider longitudinal field trials across diverse agroecological zones to evaluate agent persistence and environmental resilience. Exploring synergistic formulations that combine pterostilbene with microbial agents such as *B. subtilis* could enhance control efficacy, potentially exceeding the 70% threshold achieved in comparable pathosystems. In order to overcome the challenges outlined, researchers could adopt molecular techniques, such as qPCR for pathogen quantification and metabolomics for phytoalexin tracking, would provide more accurate and mechanistic insights into agent-pathogen interactions. To deepen our understanding of biocontrol mechanisms, future investigations might focus on greenhouse simulations of abiotic stressors, paving the way for tailored integrated pathogen management strategies in organic sorghum production and contributing to sustainable global food security. Collectively, these approaches would not only address the current limitations but also contribute to the development of sustainable crop protection practices in the face of evolving climate and disease pressures.

## 5 Conclusion

This study provides a comprehensive evaluation of several biofungicides and microbial agents against *C. sublineola*. Integrating *in vitro*, greenhouse, and field data, we found that all tested biological treatments inhibited pathogen growth and spore germination to varying extents. Iron chlorin exhibited strong antifungal activity *in vitro* but showed reduced efficacy in the field, underscoring the impact of environmental factors and formulation stability. In contrast, pterostilbene and *B. subtilis* consistently achieved superior control across environments, highlighting their potential as effective and eco-friendly alternatives to synthetic fungicides. Overall, pterostilbene and *B. subtilis* represent sustainable options for reducing chemical dependence and advancing environmentally responsible anthracnose management in sorghum production.

## Data Availability

The original contributions presented in the study are publicly available. This data can be found here: https://ngdc.cncb.ac.cn/biosample/browse/SAMC3768278.
