# Supplementary material for: Evaluation of biocontrol agents for the management of sorghum anthracnose caused by Colletotrichum sublineola
Source: Front Plant Sci. 2025 Dec 15;16:1728722. doi: 10.3389/fpls.2025.1728722 (PMC12745212; doi:10.3389/fpls.2025.1728722)
Supplement: Supplementary file 1 [file DataSheet1.docx]

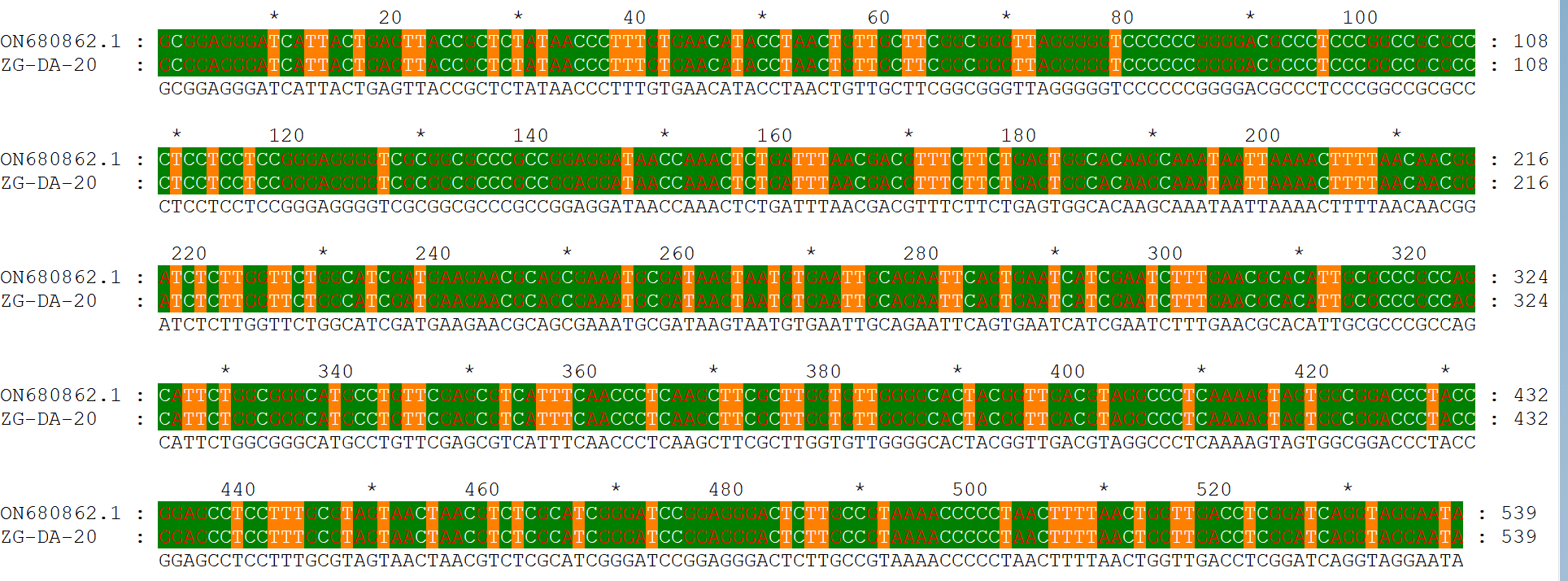


Supplementary Figure S1. Alignment of ITS sequences between strain ZG-DA-20 and ON680862.1.

Supplementary Table S1. Information on the 11 pesticides tested.

| Pesticide Name | Formulation Type | Main chemical | Content | Manufacturer |
| --- | --- | --- | --- | --- |
| Pyraclostrobin | Emulsifiable concentrate | Methoxyacrylate | 25% | BASF Plant Protection (Jiangsu) Co., Ltd. |
| Carbendazim | Suspension concentrate | Benzimidazole | 50% | Anhui Guangxin Agrochemical Co., Ltd. |
| Tebuconazole | Suspension concentrate | Triazole | 43% | Jiangsu Qizhou Green Chemical Co., Ltd. |
| S-abscisic acid | Soluble concentrate | Abscisic acid derivative | 5% | Sichuan Longmang Fusheng Technology Co., Ltd. |
| Alexin | Aqueous solution | Salicylic acid and analogs | 0.3% | Xiangyu Agricultural Technology Co., Ltd. |
| Atailing | Wettable powder | Oligosaccharide/peptide | 0.5% | Hebei Zhongbao Lunong Crop Technology Co., Ltd. |
| Iron chlorin | Soluble powder | Lipid compound | 0.5% | Anqing Baite Biological Engineering Co., Ltd. |
| HhrpEcc | Aqueous solution | Protein elicitor | 0.5% | Sichuan Haiboshi Biological Technology |
| Bacillus subtilis | Wettable powder | Microbial agent | 1×10^10^ CFU/g | Hebei Guanlong Agrochemical Co., Ltd. |
| Trichoderma harzianum | Wettable powder | Microbial agent | 2 ×10⁹ CFU/g | Hubei Qiming Biological Engineering Co., Ltd. |
| Pterostilbene | Powder | Stilbene compound | 99.5% | Beijing Solebo Technology Co., Ltd. |

**Supplementary Table S2. Concentration gradients of the 11 pesticides tested.**

| Tested Pesticide | Tested Concentration Gradient for Mycelial Growth (Spore Germination) (μg/mL) | | | | |
| --- | --- | --- | --- | --- | --- |
| Pyraclostrobin | 0.25  (0.25) | 0.4  (0.4) | 0.8  (0.8) | 2.5  (2.5) | 5  (5) |
| Carbendazim | 0.08  (0.08) | 0.17  (0.17) | 0.33  (0.33) | 0.56  (0.56) | 1.67  (1.67) |
| Tebuconazole | 0.09  （19.54） | 0.14  （23.87） | 0.29  （30.71） | 0.43  （43） | 0.86  （71.67） |
| S-abscisic acid | 6.7  (6.7) | 20  (20) | 50  (50) | 100  (100) | 200  (200) |
| Alexin | 2000  （3000） | 2400  （4000） | 3000（6000） | 4000  （15000） | 6000  （30000） |
| Atailing | （3.33） | （4.8） | （7.5） | （10） | （75） |
| Iron chlorin | 0.01  (0.01) | 0.02  (0.02) | 0.04  (0.04) | 0.08  (0.08) | 0.16  (0.16) |
| HhrpEcc | 71.43（55.6） | 100  （71.4） | 166.67（100） | 500  （166.7） | 1000  （500） |
| Pterostilbene | 50  (50) | 100  (100) | 125  (125) | 150  (150) | 200  (200) |
